# The Potential for Smart Glasses to Transform Facial Palsy Therapy Globally: UK Budget Analysis, Delphi Outcomes Valuation Exercise, and Economic Modeling of Cost-Effectiveness

**DOI:** 10.2196/67851

**Published:** 2025-11-27

**Authors:** Amir J Khan, Hema Mistry, Catriona Neville, Helen Martin, Nikki Holliday, Samuel W Oxford, Charles Nduka, Ala Szczepura

**Affiliations:** 1 Department of Economics, Institute of Business Administration, University of Karachi, Karachi, Pakistan; 2 Centre for Healthcare and Communities, Research Methods Evaluation Unit, Coventry University, Richard Crossman Building, Priory St, Coventry, CV1 5FB, United Kingdom, 44 07557425463; 3 Warwick Clinical Trials Unit and Centre for Health Economics, Warwick Medical School, University of Warwick, Coventry, United Kingdom; 4 University Hospitals Coventry and Warwickshire NHS Trust, Coventry, United Kingdom; 5 Facial Palsy Team, Queen Victoria Hospital NHS Foundation Trust, East Grinstead, West Sussex, United Kingdom; 6 Facial Palsy Service, Mersey and West Lancashire Teaching Hospitals NHS Trust, Liverpool, United Kingdom; 7 Research Centre for Physical Activity, Sport and Exercise Sciences, Coventry University, Coventry, United Kingdom; 8 Queen Victoria Hospital NHS Foundation Trust, East Grinstead, West Sussex, United Kingdom

**Keywords:** Bell palsy, facial nerve paralysis, telerehabilitation, biosensors, economic analysis, cost-effectiveness, neuromuscular retraining, patient self-management, digital rehabilitation, valuing outcome measures, economic burden, neuromuscular, quality of life, facial nerve, paralysis

## Abstract

**Background:**

Facial palsy is the most common single nerve disorder worldwide. Incidence rates are rising globally, with incomplete recovery producing long-term reductions in quality of life for one in three cases. Facial neuromuscular retraining (fNMR) to restore balanced facial function is the most widely evaluated effective nondrug therapy. There are currently no estimates of the likely economic impact of introducing telerehabilitation into the fNMR therapy pathway.

**Objective:**

This study aims to undertake an analysis of the economic burden associated with facial palsy in the United Kingdom and model the cost-effectiveness of telerehabilitation (tracking sensors in smart glasses) in the fNMR therapy pathway.

**Methods:**

The national burden associated with facial palsy was estimated including all treatment costs and economic consequences of unresolved cases. Estimates were based on annual incidence, clinical treatment patterns, recovery profiles, and impact on health-related quality of life. The monetary value placed on different levels of clinical recovery (House-Brackmann [HB] grade) was identified via a national Delphi exercise. An economic model was developed to estimate the costs and benefits of telerehabilitation from a health care perspective and to calculate incremental cost-effectiveness.

**Results:**

Direct health care costs of facial palsy treatment for all patients diagnosed each year in the United Kingdom are estimated to be £86.3 million (2020 and 2021 prices; a currency exchange rate of £1.36=US $1 is applicable). Long-term morbidity costs associated with these annual cases total £351 million to £584 million. The inclusion of societal costs, such as changes in employment, will increase this figure to over £1.27 billion. Clinical recovery from HB grades 5 and 6 is valued at >£19,400, and at £8600 for HB grades 3 and 4. Economic modeling predicts that telerehabilitation will reduce health care costs and improve outcomes, with conservative estimates indicating £468 savings per patient and a health gain of 0.14 in HB grade. If smart glasses were added to fNMR therapy for patients with incomplete recovery, this is predicted to save national health care costs of up to £3.08 million per annual cohort. The associated HB grade recovery is valued at up to £17.8 million. The inclusion of factors related to the wider societal impact (eg, on employment) will increase cost savings significantly.

**Conclusions:**

The introduction of telerehabilitation to support self-management as part of facial palsy therapy is predicted to reduce costs and improve patient outcomes, and will not require substantial early investment. Further trials with integral economic evaluations are now needed to establish cost-effectiveness in real-world settings of digitally supported fNMR early in recovery and in chronic cases.

## Introduction

A rapidly evolving ecosystem is developing in health care to provide both patients and professionals with digital solutions for disease management and rehabilitation [1]. Although evidence of the value of telerehabilitation is increasing for certain neurological disorders such as stroke, to date, there has been little consideration of facial palsy [2-4]. Bell palsy is the most common single nerve disorder worldwide, leaving patients unable to move muscles on the affected side of their face [5,6]. It is acknowledged that facial palsy is not a single entity, but rather a feature of different neurological conditions, with Bell palsy representing the majority of cases (approximately 60%) [7]. Facial palsy will affect 11‐40 people per 100,000 in the population each year, most commonly in the age group of 30‐45 years [8]. There are approximately 22,500 new facial palsy cases reported each year in the United Kingdom [9]. Occurrence can be linked to obesity, hypertension, diabetes, upper respiratory conditions, people who are immunocompromised, and pregnancy [10,11]. Although the underlying cause remains unclear [10], facial palsy has long been associated with reactivation of latent herpes virus infections [12], with rises reported to be linked to increasing rates of this infection [13]. More recently, systematic reviews have reported evidence of links with COVID-19 infections and vaccine programs [14,15]. Over the course of their lifetime, 1 in 60 people will be affected, and 30% will experience incomplete recovery [16,17]. People with incomplete recovery experience long-term reductions in quality of life, including psychological distress, depression, and social alienation [8,18-21].

Evidence of cost-effective treatments for facial palsy is limited to medication (ie, prednisolone within 72 hours of symptom onset) [22]. Among nondrug treatments, physical therapies such as facial neuromuscular retraining (fNMR) to optimize muscle resting tone and retrain balanced facial function have been most widely evaluated [23-25]. Systematic reviews provide evidence of their effectiveness early in recovery and potentially in chronic cases [9,26,27]. However, evidence on cost-effectiveness is lacking. Access to such specialist therapy is also difficult, with 1 in 10 patients in the United Kingdom reporting they travel ≥115 miles for their regular outpatient appointments [28].

Early research has suggested that biofeedback directly to patients with facial palsy could improve outcomes and reported interest among patients in innovative digital therapy or telecare [29,30]. A recent scoping review of telerehabilitation for peripheral facial palsy has identified 18 studies; these include 4 randomized controlled trial protocols, but no completed evaluations [31]. A similar scoping review of extended reality, including virtual reality (VR), has identified 7 studies but concluded that further research and validation are required [32]. Facial palsy guidelines do not currently mention telerehabilitation [24]. In contrast, the evidence base for telerehabilitation in other neurological conditions such as stroke is more well established. A systematic review of stroke home-based rehabilitation has identified over 90 existing systems, including robotics, VR, and game devices, with high effect sizes reported [33,34]. Robotic gait training is reported to produce a 52% increase in functional ambulation [35], video-guided home exercise programs have demonstrated up to 70% mobility gain [36,37], and VR use in home-based rehabilitation has shown 85% motor recovery gain [34,38]. While stroke rehabilitation guidelines currently incorporate evidence on the effectiveness of telerehabilitation [39], there still remains a significant gap in terms of any evidence on cost-effectiveness [40].

This economic analysis of telerehabilitation introduced into the fNMR pathways, therefore, addresses an important evidence gap for neurological conditions. This research is part of a wider program exploring the potential for facial remote monitoring eyewear (Frame) to transform facial palsy therapy [41]. Frame smart glasses can provide discreet feedback to patients while they undertake prescribed fNMR exercises at home and could improve monitoring by specialist therapists [42].

## Methods

### Summary

The economic analysis included three elements. First, a national budget analysis was used to assess the economic burden associated with facial palsy cases, including treatment costs and the effects of living with acquired facial palsy and long-term facial disfigurement [16]. Second, a national Delphi exercise was conducted to identify the monetary value placed on various levels of clinical recovery. Third, economic modeling was performed to assess the cost-effectiveness of adding remote monitoring eyewear to the fNMR pathway.

### Ethical Considerations

Ethics approval was granted by the Health and Life Sciences Research Ethics Committee, University of Coventry, for the surveys on UK treatment pathways and the Delphi study to evaluate outcomes (P48908). Participants formally consented. Study data were anonymous or deidentified. Participants were not provided with any compensation.

### Estimating National Treatment Costs

Medical resource use (primary care, hospital referrals, and inpatient stays) and therapy costs (fNMR and psychological therapy) were estimated per annual cohort. Estimates were based on epidemiological studies, a national survey of UK treatment pathways, and expert opinion [28,43]. Individual medical treatments were priced based on national reference costs for the 2020/2021 financial year; any earlier prices were inflated using the National Health Service (NHS) Cost Inflation Index pay and prices index [44,45]. All medical treatments were assumed to occur within 12 months following diagnosis, so no cost discounting was required. Physical and psychological therapy costs were calculated similarly.

### Estimating Annual Cohort Long-Term Morbidity Costs

The long-term morbidity associated with unresolved cases was estimated based on the annual number of UK cases, predicted recovery profiles, and reported reduction in health-related quality of life (HRQoL) for any unresolved cases [46]. For the subgroup who recover fully, HRQoL was assumed to only be affected for a period of 6 months, after which the individual returns to full health (for their age) [19]. For the subgroup with incomplete recovery, it was assumed that an individual remains in this state for the remainder of their life. The resulting reduction in HRQoL was converted to a monetary value by applying the National Institute for Health and Care Excellence (NICE) value threshold of £20,000 to £30,000 per quality-adjusted life-year [47]. A currency exchange rate of £1.36=US $1 is applicable.

### Estimating Societal Costs

It is recognized that patients with facial palsy with incomplete recovery may cease employment or move away from public-facing roles [20]. This can lead to reduced economic activity and increased social security benefit payments [48]. An approximation of these costs was based on data for people of a similar age with disabilities [49].

### Delphi Method: Valuation of House-Brackmann and Trial Outcome Measures

Trials of fNMR therapy generally report outcomes in terms of a nerve grading system, most frequently the House-Brackmann (HB) grade, rather than a utility measure (eg, EQ-5D) [9]. A Delphi exercise was undertaken over the period December 2018 to March 2019 to identify the monetary value placed on outcomes reported as an improvement in HB grade, building on earlier studies [50,51]. Purposive sampling was used to identify national experts in facial palsy and assistive technology. Invitations were sent by email, and once a panel member had consented, they were sent the round 1 Delphi questionnaire (Multimedia Appendix 1). The questionnaire was piloted for comprehension before use. In the first round, panel members were asked to place a monetary value on recovery from different grades of unilateral facial paralysis, set within monetary ranges recorded in an earlier study undertaken in the United States [50]. Consensus was set ex ante at 66% agreement. Round 1 responses were analyzed, and any statement showing consensus was extracted. In addition, panel members were provided with a list of other outcomes commonly reported in clinical trials and asked to rate their importance. Round 2 questionnaires were personalized for each panel member by presenting their round 1 scores in the context of overall panel responses; individuals could revise their ratings if they wished. The level of agreement was measured based on the modal consensus approach [52].

### Modeling of the Cost-Effectiveness of Smart Glasses Added to the fNMR Pathway

A decision analytic modeling approach was used to estimate the impact on costs and health outcomes of adding remote monitoring eyewear to the fNMR pathway [53]. The economic model was developed in Microsoft Excel and populated with pathway probabilities and health outcomes, as well as costs. The status quo was assumed to be represented by the NHS treatment pathway recorded in a UK survey [28]. It was assumed that the likelihood of patients being referred for fNMR would not be affected by the addition of telerehabilitation. The economic analysis adopted an NHS perspective and excluded patient-borne and societal costs. The cost of specialist fNMR therapy was based on a typical set of activities (units) identified by experts (physiotherapist and surgeon) and priced based on locally available data. Other health care resource items in the pathway were priced based on NHS national reference costs [44]. The cost of Frame eyewear was linked to the price identified as acceptable to UK health care providers in independent market research [54]. The average cost per pathway was estimated by multiplying each resource item by the percentage of patients referred to produce a total cost [43-45]. Prices were inflated to the 2020/2021 financial year where necessary. Health care costs in the two arms of the model were compared to differences in health outcomes (ie, improvements in HB grade). Changes in HB grade were assumed to remain the same across both arms following therapy, so that any variation in expected health outcomes results from pathway probabilities. The effect size associated with remote monitoring eyewear was based on the reported impact of telerehabilitation in other neurological conditions such as stroke and expert opinion [34]. The economic analysis followed international standards for health economics research and reporting results [55,56].

## Results

### National Treatment Costs

A breakdown of medical treatment and therapy costs is shown in Table 1. The total direct health care cost for the cohort of patients diagnosed in 2020/2021 is estimated to be £86.98 million. The majority of this figure (£86.34 million) is linked to medical costs, with 92.9% (£80.239 million/£86.339 million) associated with hospital inpatient stays, 2.7% (£2.335 million/£86.339 million) with outpatient referrals, and 4.4% (£3.765 million/£86.339 million) with general practitioner consultations and primary care prescribing. Overall, physical and psychological intervention costs are much smaller at £0.64 million, with 31.3% (£201,082/£643,292) of the total therapy figure relating to fNMR costs.

**Table 1.** UK medical treatment and therapy costs over 12 months for patients with facial palsy diagnosed annually.

Type of activity	Annual facial palsy cases, n	Unit cost (£^a^; 2020/2021 prices)	Total NHS^b^ cost (£; 2020/2021 prices)
1A: Medical treatment costs			
Primary care			
GP^c^ consultations^d^	67,971	39.00^e^	2.651 million
Corticosteroids/prednisolone^f^	22,657	45.33^g^	1.027 million
Other medication (eg, antivirals, antibiotics)^h^	5664	15.41^i^	87,282
Total primary care costs	—^j^	—	3.7653 million
Outpatient referrals			
Referral to ophthalmologist^k^	6570	123	808,110
Referral to ear, nose, and throat consultant^k^	6570	123	808,110
Referral to plastic surgeon^l^	3625	122	442,250
Referral to another specialist (eg, neurologist)^m^	2266	122	276,452
Total hospital referral costs	—	—	2.3349 million
Inpatient stay			
Total elective inpatient episodes^n^	22,457	3573^o^	80.239 million
Total medical treatment costs	—	—	86.339 million
1B: Physical and psychological therapy costs			
Facial neuromuscular retraining therapy			
Initial consultations with facial therapist^p^	1120	53^q^	59,360
Follow-up appointments^p^	2674	53^q^	141,722
Subtotal	3794	—	201,082
Referrals for other physical therapies			
Acupuncture^r^	1133	68.82^s^	77,973
Electrical stimulation^r^	1133	38.16^t^	43,235
Massage, etc^r^	1133	38.16^u^	43,235
Subtotal	3399	—	164,444
Referrals for psychological therapy			
Counselling^r^	1133	123^v^	139,359
Cognitive behavioral therapy^r^	1133	122.16^w^	138,407
Subtotal	2266	—	277,766
Total therapy costs	9459	—	643,292
Grand total treatment costs	—	—	86.982 million

a A currency exchange rate of £1.36=US $1 is applicable.

b NHS: National Health Service.

c GP: general practitioner.

d Three GP consultations per new case.

e Source: Unit Costs of Health and Social Care 2021 [45].

f All new cases treated with 50 mg for 10 days.

g Source: NICE Clinical Knowledge Summaries [57].

h 25% of cases treated with 75 mg twice daily of oseltamivir.

i Source: NICE British National Formulary [58].

j Not applicable.

k One referral per patient not fully recovered (29%).

l All patients with permanent deficit (16%).

m 10% of new cases referred for expert opinion.

n Total recorded for patients with Bell palsy in 2016/2017 (scaled up for facial palsy cases).

o Source: Cooper et al [43].

p Survey of national specialist centers [28].

q Physiotherapist/occupational therapist band: 60 minutes per session [45].

r 5% of new cases.

s NHS Schedule of Reference costs for years 2019/2020 (adjusted to 2020/2021 prices) [59].

t Fees paid at private clinic (adjusted to 2020/2021 prices).

u Nonspecialist rehabilitation services level 3, NHS reference costs [59].

v Source: Unit Costs of Health and Social Care 2021 [45].

w Therapy sessions (£173/session, £14 per service user; uplifted to 20/21 prices) [60].

### Annual Cohort Long-Term Morbidity Costs

The long-term morbidity burden associated with unresolved cases per annual cohort is shown in Table 2 in terms of quality-adjusted life-years loss. After applying the NICE threshold, this long-term loss is valued at between £238 million and £357 million per cohort. This is 2.7 to 4.1 times the estimated total medical and therapy treatment cost of £86.98 million. The low cost of fNMR therapy in Table 1 partly reflects the fact that only 4.9% (1120/22,657) of new cases or 17.0% (1120/6570) of unresolved cases are reported to be referred for fNMR.

**Table 2.** Annual number of cases, recovery profiles, quality-adjusted life-years lost, and morbidity cost.

Level of recovery	Annual facial palsy cases^a^, n (%)	Time in health state	Total QALYs^b^ lost^c^	Morbidity cost (£ millions^d^)
Full recovery	16,086 (71.0)	2-3 weeks to 9 months (average 6 mo)	322	6.44-9.60
Partial recovery	2945 (13.0)	44.1 years^e^	5195	103.9‐155.85
Permanent deficit	3625 (16.0)	44.1 years^e^	6395	127.9‐191.86
All cases diagnosed	22,657 (100.0)	—^f^	11,912	238.24‐357.31

a Based on Bell palsy incidence (scaled up to all facial palsy cases) plus reported recovery pattern [8,61,62].

b QALY: quality-adjusted life-year.

c Based on average reported decrease in health-related quality of life [46].

d A currency exchange rate of £1.36=US $1 is applicable.

e Based on median age of Bell palsy diagnosis (37.5 y) and average length of life in the United Kingdom (81.6 y) [28].

f Not applicable.

### Societal Costs

Incomplete recovery can lead to reduced economic activity, social security benefit payments, and loss of tax revenues generated through work [48]. Such societal costs are not included in Table 1. However, people of a similar average age (40 y) with 10% to 20% disablement are reported to receive cumulative social security benefit payments of £5000-£10,000 over 5 years [49]. Applying this to a facial palsy cohort would add a further societal cost of £113.3 million to £226.6 million over 5 years. This produces a final conservative estimate for the total economic burden, including societal costs, of £351 million to £584 million per annual cohort.

### Delphi Valuation of HB Grade and Other Trial Outcome Measures

All 26 experts invited to join the Delphi panel accepted (see Multimedia Appendix 1 for panel details). All panel members completed the round 1 questionnaire, with 19 (73.1%) returning the round 2 questionnaire. Table 3 shows the values placed on a patient’s clinical recovery from different HB grades of paralysis and ratings of the importance of other outcomes reported in trials. The modal rating for high grades of 5 and 6 paralysis achieved predefined consensus (66% agreement) in round 1 with 83% agreement, and for medium severity grades of 3 and 4 paralysis in round 2 with 69% consensus. The modal value for low-severity paralysis failed to show consensus by the end of round 2 (35% agreement). Multimedia Appendix 2 provides a description of HB grades.

Table 3 also shows the perceived importance of other outcomes most commonly reported in evaluation studies. There was full consensus (100%) on four measures rated as being “very important,” with three further outcomes (psychological distress, patient-borne costs, and NHS treatment costs) achieving a lower level of agreement at 88%‐96%, but well above the ex ante 66% level required for consensus. The one outcome rated “important” (impact on employment) demonstrated consensus at 77% agreement.

**Table 3.** Delphi panel consensus on key outcome measures and value placed on level of recovery (2019/2020 prices).

Statements	Agreement (%)^a^	Modal rating (when consensus was reached)
Repair of House-Brackmann grades		
High-grade paralysis (grades 5 and 6)	83	≥£19,400^b^ (round 1)
Medium-grade paralysis (grades 3 and 4)	69	≥£8600 (round 2)
Low-grade paralysis (grades 1 and 2)	35	≤£1800
Key outcome measures		
Appearance/facial symmetry^c^	100	Very important (round 1)
Facial paralysis/motor recovery^c^	100	Very important (round 1)
Pain/facial discomfort^c^	100	Very important (round 1)
Social function^c^	100	Very important (round 1)
Psychological distress	96	Very important (round 1)
Patient-borne costs (eg, travel)	92	Very important (round 1)
National Health Service treatment costs	88	Very important (round 1)
Change of employment	77	Important (round 1)

a Percent agreement was based on the modal consensus approach [52].

b A currency exchange rate of £1.36=US $1 is applicable.

c These outcome measures were all equally ranked as first.

### Cost-Effectiveness of Smart Glasses Added to fNMR Pathway

**Figure 1.**
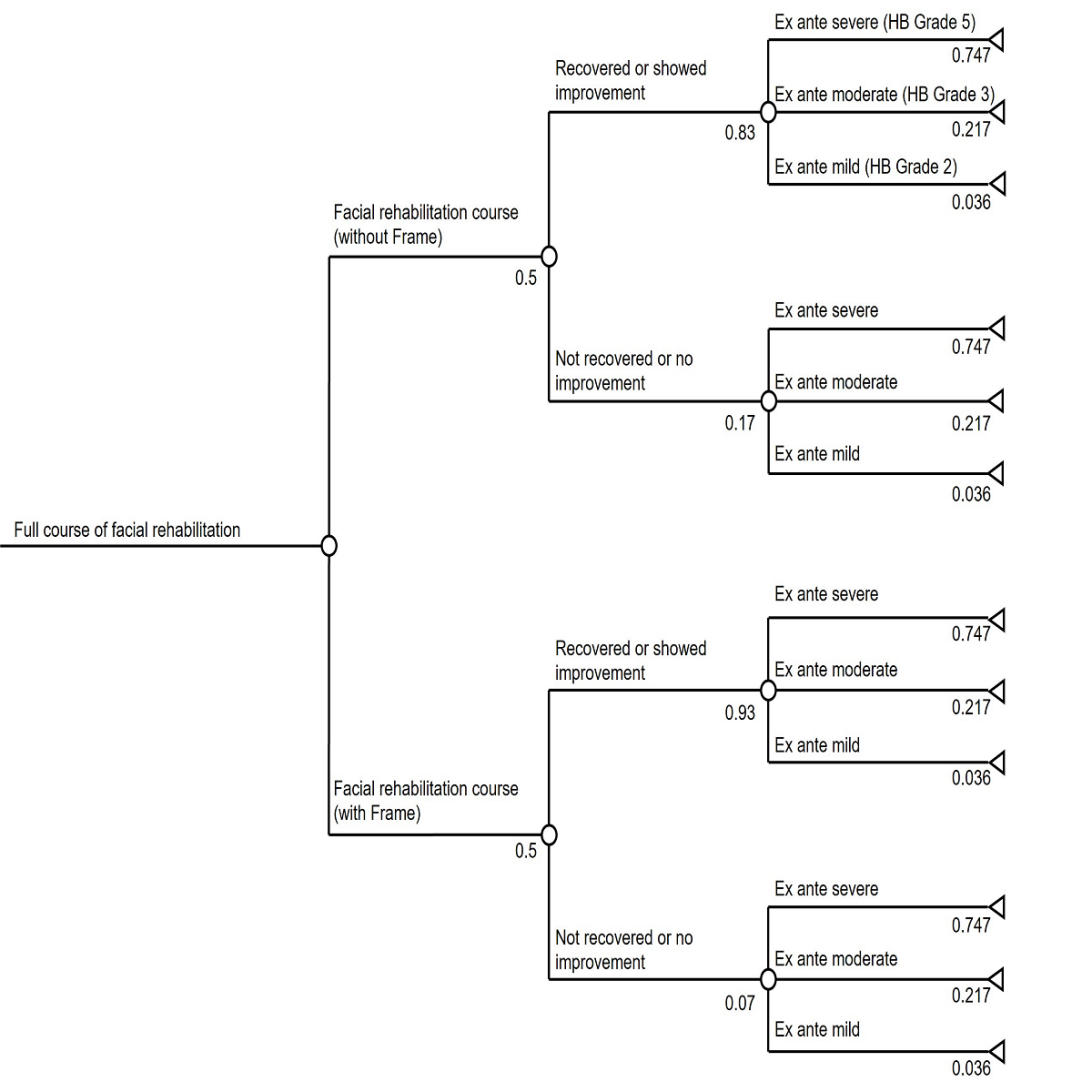
Decision analytic model structure: facial neuromuscular retraining with and without telerehabilitation. HB: House-Brackmann.

A decision tree model was constructed as shown in Figure 1. An initial node represents patients who do not recover fully in the first 6 months following initial treatment (eg, prednisolone and advice on eye care). From this initial node, there are two branches: the face-to-face fNMR pathway (status quo) and telerehabilitation (Frame pathway). Each branch then develops two arms: one resulting in no further recovery and one showing some or full recovery. There are then three further branches depending on the state of facial paralysis at entry level (ie, HB grades severe, moderate, or mild). The chance of recovery with telerehabilitation, compared to the status quo, was set at the lowest effect sizes reported for telerehabilitation introduced into stroke pathways [34]. Probability-weighted costs and outcomes were calculated for the two pathways separately, after adjusting corresponding absolute values with joint probabilities of events along the pathway.

Table 4 presents a bottom-up cost for a full fNMR pathway (status quo). In addition to fNMR appointments, the total cost includes an initial multidisciplinary assessment and administration of botulinum toxin injections. As a check, the final estimate calculated in this way was then compared to contract prices recorded by service commissioners in England and found to fall well within the range reported [63]. The unit price of the eyewear was set at a high figure of £495.83 (175% of the average £283.33 reported to be an accepted price point in the market survey) [54].

**Table 4.** Total cost of the full course of facial neuromuscular retraining (fNMR) therapy (2020/2021) prices^a^.

Status quo activity	Unit cost (£)^b^	Units	Total cost per patient (£)
Initial multidisciplinary team clinic appointment	159.68	1	159.68
Psychological assessment	95.72	1	95.72
fNMR appointments (with neurophysiotherapist)	65.85	6	395.10
Botulinum toxin appointments	247.57	10	2475.65
Total cost of fNMR per patient	—^c^	—	3126.10

a Source: Unit Costs of Health and Social Care 2021 [45].

b A currency exchange rate of £1.36=US $1 is applicable.

c Not applicable.

Table 5 presents the incremental cost-effectiveness ratio analysis. This indicates that a full course of fNMR incorporating smart glasses will both cost less and result in better health outcomes than the status quo. In other words, the pathway including Frame eyewear is “dominant.” Per patient, the cost saving is estimated to be £468, and the health gain an improvement of 0.14 in HB grade per person.

**Table 5.** Incremental cost-effectiveness ratio analysis (probability-weighted costs and outcomes).

Item	Expected cost of facial therapy (£)^a^	Expected health gain (House-Brackmann grade)
With Frame	1095.38	1.261
Without Frame	1563.07	1.125
Incremental difference	–467.69	+0.14

a A currency exchange rate of £1.36=US $1 is applicable.

## Discussion

### Principal Results

To our knowledge, this is the first study to assess the economic burden associated with facial palsy, including the costs of treatment and impact on HRQoL, and to model the cost-effectiveness of introducing telerehabilitation into the fNMR pathway. Our economic modeling shows that if remote monitoring (tracking sensors in smart glasses) were to be added to the current pathway, this would prove “dominant” from a health care perspective. In other words, telerehabilitation is predicted to both reduce overall health care costs and result in better outcomes for patients. Scaled up to a national level, the cost saving predicted per patient in the base case would result in savings of up to £3.08 million if all 6570 patients with incomplete recovery in an annual UK cohort were offered digitally supported fNMR, or £0.52 million if it were limited to unresolved cases currently referred for fNMR. Furthermore, according to the Delphi panel valuation, the health gain in terms of HB grade improvement for 6570 patients would represent up to £17.8 million. Within a context where the economic burden associated with residual deficits in unresolved cases is £238 million-£357 million per annual cohort, there is considerable room for investment in technologies that could improve long-term quality of life [16]. Changes in employment for unresolved cases will increase long-term costs to £1.27 billion [48,64], offering even more potential if an innovation can reduce societal costs by improving patient outcomes. In the context of a rise in facial palsy cases [13-15,65-68], it is even more important to identify and evaluate such innovations.

### Limitations

There are a number of limitations to our economic evaluation. First, the inputs used in the decision tree model are based on average costs and outcomes, while studies indicate that treatment and recovery patterns can vary across patients [28,69]. Ideally, a future model should incorporate such heterogeneity. Second, our economic model used HB grade improvement as the outcome measure since this is most commonly used in studies of effectiveness [9]. However, we were reliant on data from stroke trials for an estimate of the effect size associated with the introduction of telerehabilitation, so the model could not fully account for different levels of HB grade or severity. Because of this uncertainty, a conservative effect size was used, far lower than the 52% to 85% levels reported for stroke [34-36]. In terms of cost inputs, the economic model also set a high price for the wearable device at £496 or 175% of the average acceptable price reported in independent market research [54]. In fact, because incorporating smart glasses reduces health care costs and improves health outcomes, the cost of the device could be increased further up to a break-even price of £964 (US $1234) and still prove cost-effective. The break-even price falls between that of Meta Ray-Ban smart glasses (US $299), which offer artificial intelligence–assisted entertainment [70], and the Apple Vision Pro (US $3499), which provides mixed reality experiences [71]. The unit cost assumed single-use, but Frame devices could be reused by a health care provider, lowering unit costs [72]. A pathway with digital support might also reduce the number of certain costly treatments associated with fNMR (eg, botulinum toxin appointments). The model made no such assumption. Finally, because our analysis adopted a health care perspective, this means that patient costs are excluded, although some, such as patient-borne travel costs for regular outpatient appointments, can be substantial [28]. The impact on a person’s income associated with changes in employment for unresolved cases was also excluded but can be significant [18,21].

In terms of outcomes, our economic model may underestimate benefits. First, it did not consider the possibility of increased adherence to prescribed fNMR exercises, although there is some evidence of this [29]. Second, it assumed no impact related to accurate monitoring by clinicians, although there is evidence that professionals assume better adherence levels than those recorded by patients themselves [28]. For stroke, there is international evidence of wide variations in the monitoring of rehabilitation [73]. Third, telerehabilitation at an earlier point might also result in less entrenched dyskinetic patterns and, in some cases, could minimize more severe residual involuntary movements known as synkinesis [8]. People with these severe residual deficits experience a greater long-term reduction in quality of life [8,18-21].

In summary, although the data did not allow a full sensitivity analysis to be undertaken, the assumptions made about costs and effectiveness in this preliminary economic model are conservative and probably underestimate real-world benefits and cost savings. Ideally, a lifetime horizon should be explored in future economic analyses. It should also be borne in mind that the model is based on UK fNMR pathways, but these may vary internationally.

### Comparison With Other Work

UK guidance on the management of facial palsy currently includes no mention of telerehabilitation or consideration of its cost impact or likely cost-effectiveness [24]. An evidence gap remains, even though a study of the implications of telemedicine published in 2019 recommended that future research should consider costs [74]. This study is part of an ongoing research program assessing the value of adding telerehabilitation to the facial palsy fNMR pathway. This includes a market assessment to explore pricing and routes to market [54], a national survey of UK treatment pathways [28], and a systematic review to update evidence on fNMR effectiveness now added to NICE guidelines on management of facial palsy [9,24]. Economic evaluations similar to this study are limited. There is no mention of facial palsy in a general review of telerehabilitation [2], two systematic reviews of economic analyses of home-based telerehabilitation [4], or an economic evaluation of physiotherapy interventions for neurological disorders [3]. For severe neurological conditions, an evaluation reports that remote physical rehabilitation may cost less and be more effective in the mildest cases [75]. For stroke, a Cochrane review found evidence of improved outcomes, although limited evidence on cost-effectiveness [76]. A more recent NICE evidence review has reported that stroke telerehabilitation delivered as an adjunct and telerehabilitation delivered alone are equally effective, but found little research on cost-effectiveness [40]. An earlier study of VR-based stroke telerehabilitation did report reduced costs (£457 less) but no difference in balance recovery [77]. Another VR study only reported equipment implementation costs and did not consider wider health care costs or outcomes [78].

### Conclusions

Long-term morbidity and societal costs associated with facial palsy are estimated to be £351 million to £584 million per annual cohort, indicating significant possible savings if long-term recovery can be improved. Economic modeling confirms that the addition of telerehabilitation to fMNR could improve patient outcomes and reduce costs when compared to current in-person therapy. Because access to specialist neurophysiotherapy services is limited in the United Kingdom, the introduction of telerehabilitation could also enable increased access for currently underserved populations. Further trials with integral economic evaluations in real-world settings are now needed to establish the cost-effectiveness of digitally supported fNMR both early in recovery and in chronic cases. Such studies should meet evidence standards for digital health technologies [79]. If both clinical (HB grade) and utility (EQ-5D-5L) outcomes are included, this covers the top five outcomes identified by the Delphi panel in this study.

Any future investment in telerehabilitation to support home-based fNMR therapy will need to be driven both by specialist facial therapists and by patients. As with other health care innovations, implementation is likely to meet organizational and cultural barriers [80], often reinforced by policy priorities [81]. Although we have drawn on evidence from stroke telerehabilitation, similar economic analyses are lacking, including for facial palsy following stroke [82]. However, implementation of telerehabilitation for stroke does highlight certain challenges for policy makers, including potential exclusion of some patients, the need to address staff training, and awareness of the existence of variable practices [83]. In terms of digital exclusion, there is a need to ensure that implementation does not further exclude patients who require services [84]. Training in fNMR is essential because a shortage of trained neurophysiotherapists is leading to many general therapists delivering the service; the development of international online specialist training should help address this issue [85], together with international guidelines to reduce any variations in practice [86]. However, further training will be required for the successful introduction of a digital service since interaction online will differ from face-to-face clinical consultations [87]. For stroke, there are currently no published standards or guidelines for telehealth, and wide variations are reported in quality and monitoring practices [73]. At a time when 26 of 32 European countries are in favor of implementing home rehabilitation for stroke [88], recommendations on the organization of such a service have only recently been published [89]. Any future implementation of telerehabilitation for facial palsy should be considered in light of international developments for neurological services more widely. Although based on UK data, the findings reported here should be of interest in this international context.

## Supplementary material

10.2196/67851 Multimedia Appendix 1 Delphi questionnaire and panel details.

10.2196/67851 Multimedia Appendix 2 House-Brackmann Outcome Scale.

10.2196/67851 Checklist 1 CHEERS 2022 checklist.

## Abbreviations

fNMR: facial neuromuscular retraining
HB: House-Brackmann
HRQoL: health-related quality of life
NHS: National Health Service
NICE: National Institute for Health and Care Excellence
VR: virtual reality
