# Supplementary material for: The Potential for Smart Glasses to Transform Facial Palsy Therapy Globally: UK Budget Analysis, Delphi Outcomes Valuation Exercise, and Economic Modeling of Cost-Effectiveness
Source: J Med Internet Res. 2025 Nov 27;27:e67851. doi: 10.2196/67851 (PMC12661599; doi:10.2196/67851)
Supplement: Multimedia Appendix 1 [file jmir-v27-e67851-s001.pdf]

1. Delphi Questionnaire

Delphi Expert Panel:

Telecare Innovation for Facial Nerve Palsy Therapy

ROUND 1

We would like to thank you for agreeing to join our Delphi panel to help us identify key aspects important for any future decisions about the introduction of Facial Remote Activity Monitoring Eyewear (described below).

Our goal is to canvas national opinion on the key measures or data required to evaluate the introduction of this innovation from the perspectives of the medical community generally, facial exercise therapists, patients and CCGs/health commissioners.

At the heart of most evaluations of a healthcare technology lies the need to consider the added benefits provided (if any) versus any resource or cost consequences. There may also be organisational implications, as well as other potential consequences, which need to be considered in a health technology assessment.

BACKGROUND INFORMATION

Facial palsy

In the UK approximately 22,500 new facial nerve palsy cases occur each year. This means that there have been over one-third million cases since 2000. Although seventy per cent of these cases will achieve complete recovery, it is estimated that 115,000 people diagnosed since 2000 are still living life with some level of disability. This includes 63,500 with a permanent deficit of facial function. Over a lifetime this deficit can result in a loss of 2 quality adjusted life years (QALYs) per patient. Recent US epidemiological studies report a 65% rise in facial nerve (Bell's) palsy cases over the period 1968 to 2010, possibly linked to increasing rates of herpes zoster infection.

Facial rehabilitation exercise therapy

Various treatments are available for facial nerve palsy. Among non-medical treatments, tailored facial exercise therapy (designed to increase muscle and improve nerve function) has the largest evidence base. Although there are variations in practice and how recovery is measured, facial exercise therapy generally consists of:

1. initial outpatient appointment with specialist therapist for assessment and tailored exercise prescription
2. further outpatient visits (up to 18) to monitor progress and deliver additional therapy, with the longest distances travelled averaging 350 miles across English regions

3. patient daily exercise (self-massage, stretching and neuromuscular retraining) approx. 30 minutes
4. continued daily facial exercises until a recovery plateau is reached (approx. 1-2 years)

Facial Remote Activity Monitoring Eyewear (FRAME)

FRAME is a novel, cross-platform system designed to improve facial rehabilitation. The system (see diagram below) includes:

- FRAME mask for home-based facial electromyography (EMG). EMG is an established technique which can provide real-time biofeedback on facial muscle activity to patients and therapists to ensure patients practice their exercises correctly

- FRAME glasses with miniaturised sensors embedded in the frames. These can be worn throughout the day to monitor facial expression and transmit data via an app to the patient's smartphone, tablet or PC. Data can also be sent securely via the cloud to a specialist therapist. The principal outputs are muscle activity, left and right balance and some visual representation of eye closure and smile

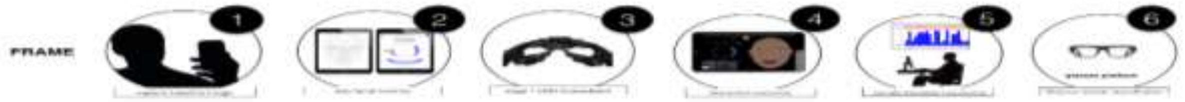

Because people with facial paralysis have a very limited awareness or control of any abnormal facial movements, they currently practice their facial exercises in front of a mirror. But many dislike seeing their own reflection as it reminds them of their condition, and can make them feel discouraged and stop exercising altogether. FRAME will allow patients to practice exercises discreetly while going about their lives and provide them with information on their progress. Rather than spending short periods of time exercising, their rehabilitation can become part of their day-to-day interactions.

The patient's smartphone will provide a schedule of routines, give live feedback, data on muscle tone, number of repetitions, weekly progress and historical information.

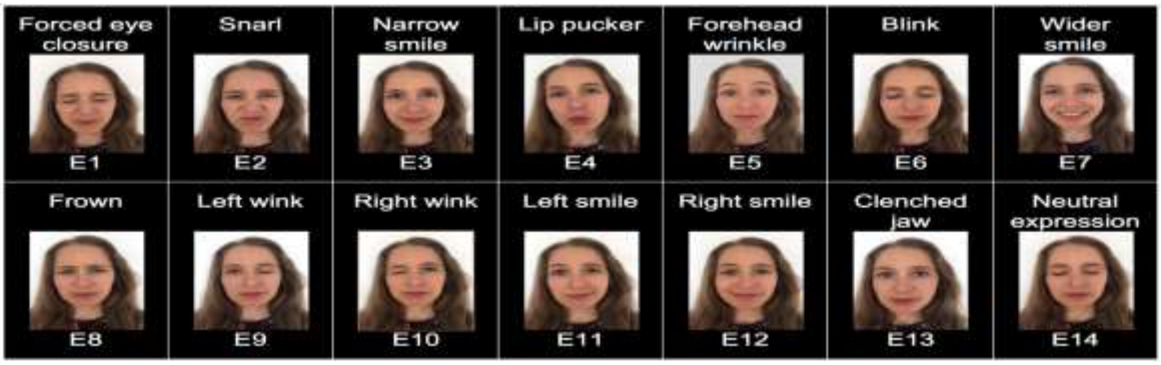

Technology assessment framework

On the basis of discussion with a number of experts, a published international survey on the potential for mobile technology to transform healthcare, and our own systematic review of the effectiveness of

facial exercise therapy in Bell's palsy we have identified several distinct aspects which it might be important to consider in any future assessment of this tele-rehabilitation technology.

Delphi Exercise

In this first round of the Delphi exercise, we ask you to rate your agreement with various general statements in relation to the FRAME technology. This will enable us to rank these aspects according to their importance to any decision on the introduction of this particular form of telecare. We then ask you to consider various possible outcome measures and rate the importance of these. Finally, you are asked to place a value on recovery from unilateral facial paralysis of three severity levels.

In round two, you will receive anonymised analysis of all rounds 1 responses and an amended list for further comment and revision. A final list of agreed, ranked, and categorised responses will be developed based on median scores. We anticipate two Delphi rounds.

Timescale for Delphi exercise

In order that we may analyse the responses of this round promptly and circulate the second round of the Delphi exercise as soon as possible, we would be most grateful if you could **complete your questionnaire within two weeks of opening this email.**

A pilot indicates that this first questionnaire should take approximately 10 minutes to complete.

A second round questionnaire, including an analysis of everyone's responses, will be sent to you **in early November**. This will enable us to produce a **preliminary report by early December**, which will be circulated to all Panel members.

When you registered to take part, we asked you to generate a unique 6-digit code using your date and month of birth plus your initials. *(e.g. for 13th February and name Joan Smith this is 1302JS).*

Please enter your 6-digit code below so we know you have taken part in Round 1.

**YOUR ID**						

Q1: Do you agree with the following positive outcomes suggested for introduction of FRAME technology?

For each statement, please rate your level of agreement on a scale of 1-5 (1 = 'Very low agreement' and 5 = 'Very high' agreement).

Please add any further positive outcomes you identify as important, together with your rating (1-5).

Rating (1-5)	Positive outcomes
	Greater patient access to data will encourage people to take control over their own care and recovery
	Operational stresses on healthcare organisations will decrease
	Patients' day-to-day adherence to facial exercises will be improved
	Reduced patient costs (e.g. by providing less expensive way to access healthcare)
	Costs for institutions will fall
	Help healthcare providers to target underserved communities (e.g. in rural areas)
	Improve communication between providers and the patients they serve
	I do not think that FRAME technology will have a noticeable effect on outcomes
	Other<br>*Please specify*
	Other<br>*Please specify*

Q2: Do you agree that the following are currently the biggest challenges for patient adoption of FRAME technology?

For each statement, please rate your level of agreement on a scale of 1-5 (1 = 'Very low agreement' and 5 = 'Very high' agreement).

Please add any further challenges to patient adoption you identify as important, together with your rating (1-5).

Rating (1-5)	Challenges to patient adoption
	Consumers and/or patients are not used to the idea of tech-based health services
	Consumers are wary of their data privacy being violated
	These technologies are too complicated (e.g. for the ill or elderly)
	Consumers and/or patients suspect the devices are not reliable
	Mobile services are not appealing enough
	I do not think there are any major challenges to patient adoption of FRAME technology
	Other<br>*Please specify*
	Other<br>*Please specify*

Q3: Which of the following do you think are currently the biggest challenges for NHS adoption of FRAME technology?

For each statement, please rate your level of agreement on a scale of 1-5 (1 = 'Very low agreement' and 5 = 'Very high' agreement).

Please add any further challenges to NHS adoption you identify as important, together with your rating (1-5).

Rating (1-5)	Challenges to NHS adoption
	Institutional bias and conservatism within the healthcare establishment
	The need to ensure patient privacy
	Therapists are not sufficiently technologically sophisticated to be able to draw on this mobile technology
	Concerns about burdensome technological complexity and continual updates
	Transitioning to new technology will cost too much
	Lack of wireless or spotty phone-network coverage will impair access (e.g. in rural and/or disadvantaged areas)
	The need to accommodate many user needs (e.g. multiple languages, disabilities)
	I do not think there are any major challenges to NHS adoption of FRAME technology
	Other<br>*Please specify*
	Other<br>*Please specify*

Q4: Do you agree with the following concerns about the FRAME technology and its introduction?

For each statement, please rate your level of agreement on a scale of 1-5 (1 = 'Very low agreement' and 5 = 'Very high' agreement).

Please add any further concerns regarding introduction of the FRAME technology you identify as important, together with your rating (1-5).

Rating (1-5)	Concerns regarding technology introduction
	People may misinterpret their own data and make poor decisions
	Data privacy risks
	Legal risks
	People may get poor information and make poor decisions
	Potential additional cost for individuals
	Potential additional cost for institutions
	People will feel self-conscious/embarrassed about having wearable devices
	I do not have any concerns about introduction of FRAME technology
	Other<br>*Please specify*
	Other<br>*Please specify*

Q5: How important is it measure the following outcomes when evaluating FRAME technology?

For each statement, please rate your level of agreement on a scale of 1-5 (1 = 'Very low agreement' and 5 = 'Very high' agreement).

Please add any further outcomes which you consider important for evaluation of FRAME technology, together with your rating (1-5).

Rating (1-5)	Outcomes
	**Personal Outcomes**
	Appearance/ facial symmetry
	Facial paralysis/ motor recovery
	Pain/ facial discomfort
	Psychological distress
	Social function
	Change of employment
	**Resource Impact**
	NHS treatment costs
	Patient-borne costs (e.g. travel)
	Other<br>*Please specify*
	Other<br>*Please specify*

Q6: A general population sample in the USA has recently placed a value on recovery from unilateral facial paralysis of 3 severity levels (low, medium, and high) categorized by House-Brackmann grade.

Low = House-Brackmann grade 2.

Medium = House-Brackmann grades 3 and 4.

High = House-Brackmann grades 5 and 6.

Please place your own valuation on repair of each grade of paralysis (tick <u>one value only</u> per grade). There is no right or wrong answer:

Low-grade paralysis

- Less than £1,800 – please specify
- £1,800
- £2,700
- £3,800
- More than £3,800 – please specify

Medium-grade paralysis

- Less than £4,900 – please specify
- £4,900
- £6,600
- £8,600
- More than £8,600 – please specify

High-grade paralysis

- Less than £12,500 – please specify
- £12,500
- £15,700
- £19,400
- More than £19,400 – please specify

YOUR DETAILS

We would be grateful if you could please provide some details about yourself.

The following information is important in order to develop an aggregate profile of Delphi Panel members. This will be treated in strict confidence.

1. Your background (please tick only one descriptor)

Tick only one	Background
	Patient or relative of someone with facial palsy
	NHS commissioner
	Facial therapy specialist
	Hospital medical specialist
	General practitioner
	Academic with an interest in facial palsy
	Other – please specify

2. How many years' experience of facial palsy do you have?

- ❑ Years

3. Does this include familiarity with facial exercise therapy?

Yes/No

**If Yes**, please describe briefly:

If you have any additional comments, we would be very pleased to hear them.

Thank you for taking the time to complete our survey.

2. Delphi Panel Members: Reported Characteristics*

Description	How many patients seen in an average month?	Number years' experience of facial palsy	Familiarity with facial neuromuscular retraining (facial exercise therapy)
Patient or relative of someone with facial palsy	N/A	49	Yes
Patient or relative of someone with facial palsy	N/A	3	Yes
Patient or relative of someone with facial palsy	N/A	3	Yes
Facial therapy specialist	100	25	Yes
Hospital medical specialist	200	12	Yes
Hospital medical specialist	30	25	Yes
Hospital medical specialist	16	16	Yes
General practitioner	160	36	No
Facial therapy specialist	40	11	Yes
Other - Academic with interest in digital technologies	N/A	N/A	N/R
NHS commissioner	0	0	No
Physiotherapist	20	5	Yes
General practitioner	200	40	No
General practitioner	130	30	No
Other - Academic with interest and expertise in telehealth - particularly from the patient / service user perspective	Zero	0	N/R
Other - NHS Commercial and Intellectual Property Manager	None	0	No
Other - Health Technology Assessment	N/A	0	No
Patient or relative of someone with facial palsy	N/A	2	Yes
Other - Director Council for AHP research	N/A	0	N/R
Occupational therapist	0	0	No
General practitioner	0	25	No
Hospital medical specialist	45	12	Yes
Hospital medical specialist	250	5	No
Patient or relative of someone with facial palsy	N/A	2	Yes
Physiotherapist	5	2	Yes
Facial therapy specialist	25	29	Yes
Physiotherapist	20	3	Yes

* 24 / 26 round 1 respondents provided details.
