# Supplementary material for: The Potential for Smart Glasses to Transform Facial Palsy Therapy Globally: UK Budget Analysis, Delphi Outcomes Valuation Exercise, and Economic Modeling of Cost-Effectiveness
Source: J Med Internet Res. 2025 Nov 27;27:e67851. doi: 10.2196/67851 (PMC12661599; doi:10.2196/67851)
Supplement: Multimedia Appendix 2 [file jmir-v27-e67851-s002.pdf]

Description

The House-Brackmann scale is a nerve grading system developed to characterize the severity of a facial paralysis patient's symptoms. The score is determined based on a measurement of the upward movement of a facial paralysis patient's eyebrow and the outward movement of the mouth. All scores are made based on eyebrow and mouth movement on the non-paralyzed side of a patient's face

House-Brackmann (HB) Grades

House-Brackmann classification scores include the following:

- **Grade I:** Normal
- **Grade II:** Slight facial weakness or other mild dysfunction. Normal tone and symmetry at rest. Complete closure of the eye without effort. Slight asymmetry of the mouth when facial movements occur.
- **Grade III:** Assigned to patients dealing with moderate dysfunction; these patients generally do not display any noticeable facial weakness with synkinesis, they maintain complete eye closure and good forehead movement with effort.
- **Grade IV:** Assigned to patients dealing with severe dysfunction. Obvious facial weakness. Incomplete eye closure, no forehead movement, asymmetrical mouth movement, and synkinesis.
- **Grade V**: Assigned to patients who have little to no ability to smile, frown or make other facial expressions. The closure of the eye is incomplete, and there is no forehead movement.
- **Grade VI**: No facial motion.

Validation

House JW, Brackmann DE. Facial nerve grading system. *Otolaryngol Head Neck Surg*. 1985;93:146–147

Languages

The House-Brackmann scale is not dependent on language spoken.
